# A New Positioning Method for Climbing Robots Based on 3D Model of Transmission Tower and Visual Sensor

**DOI:** 10.3390/s22197288

**Published:** 2022-09-26

**Authors:** Yansheng Liu, Junyi You, Haibo Du, Shuai Chang, Shuiqing Xu

**Affiliations:** School of Electrical Engineering and Automation, Hefei University of Technology, Hefei 230009, China

**Keywords:** positioning method, climbing robot, transmission tower

## Abstract

With the development of robot technology and the extensive application of robots, the research on special robots for some complex working environments has gradually become a hot topic. As a special robot applied to transmission towers, the climbing robot can replace humans to work at high altitudes to complete bolt tightening, detection, and other tasks, which improves the efficiency of transmission tower maintenance and ensures personal safety. However, it is mostly the ability to autonomously locate in the complex environment of the transmission tower that limits the industrial applications of the transmission tower climbing robot. This paper proposes an intelligent positioning method that integrates the three-dimensional information model of transmission tower and visual sensor data, which can assist the robot in climbing and adjusting to the designated working area to guarantee the working accuracy of the climbing robots. The experimental results show that the positioning accuracy of the method is within 1 cm.

## 1. Introduction

The high-voltage transmission network is crucial and indispensable for large-scale energy transmission, in which the transmission tower plays an important role in carrying high-voltage transmission lines and maintaining safe distances [1]. The maintenance and assembly of transmission towers are dangerous and arduous tasks that rely on manual work for a long time [2]. There are disadvantages such as the high safety risks for operators, harsh field operation environment, and low degree of automation. In recent years, with the rapid development of robot technology, the research and development of robots to automatically carry out the maintenance and assembly of transmission towers have attracted the attention of more and more researchers. In Ref. [3], a semiautomatic construction manipulators system for power transmission towers was proposed. The operation and control methods were expounded, and a fundamental concept for the construction manipulator system of a transmission tower was established. In Ref. [4], a power transmission tower climbing robot consisting of two upper and lower clamping mechanisms and a linear motion mechanism was designed, and the feasibility of the clamping mechanism was preliminarily verified by the prototype development and experiments. In Ref. [5], an auxiliary climbing robot for the maintenance of a transmission tower was developed to reduce the safety risk of operators.

At present, most of the research on climbing robots for transmission towers is concentrated on structural design and system construction, and there are still many difficulties to be solved before mature applications. Among them, the positioning problem is the basis for precise navigation and autonomous operation. The robot positioning methods can be mainly divided into two types: relative positioning methods and absolute positioning methods [6]. Relative positioning methods mainly use sensors, odometers, and other hardware devices to measure the relative position and attitude changes during robot motion [7]. Absolute positioning methods complete the global positioning by using fixed beacons in the working scene [8]. For the relative positioning methods, navigation is mostly completed through the inertial measurement unit (IMU). The accuracy of relative positioning depends on the accuracy of the sensors and can be disturbed by noise. The absolute positioning methods for robots include beacon positioning method [9], SLAM method [10], and other positioning methods. The beacon positioning method is mainly by arranging beacons in a fixed position of the environment in advance, and then acquiring the position information by using sensors to detect the beacons.

The beacon positioning method has obvious advantages in complex environments. In Ref. [11], a visual navigation and path tracking method (SGI) of UAVs was presented using the geometric information and landmark information of street images without relying on GPS. A new implementation method (VRL) for indoor environment representation and visual relocalization using RGB-D sensor was presented in Ref. [12]. Using regression forest, landmark positions were efficiently predicted and the camera poses were then estimated in a probabilistic framework. A positioning method (CVIS) based on a visual and inertial sensor combination was presented in Ref. [13], by which the navigation accuracy of the indoor mobile robot was improved and error accumulation was eliminated.

In order to complete precise positioning of a climbing robot on the transmission tower, this paper proposes an intelligent positioning method that integrates the 3D information model of the tower and the visual sensor data. The method obtains the position information of the bolt feature points as beacons by deconstructing the accurate 3D information model of the transmission tower, and matches it with the visual sensor data. The experiment results show that the accuracy of the proposed positioning method is within 1 cm.

## 2. Analysis of the Tower Information Model

### 2.1. Analysis of the Working Environment of a Transmission Tower

**Figure 1:**
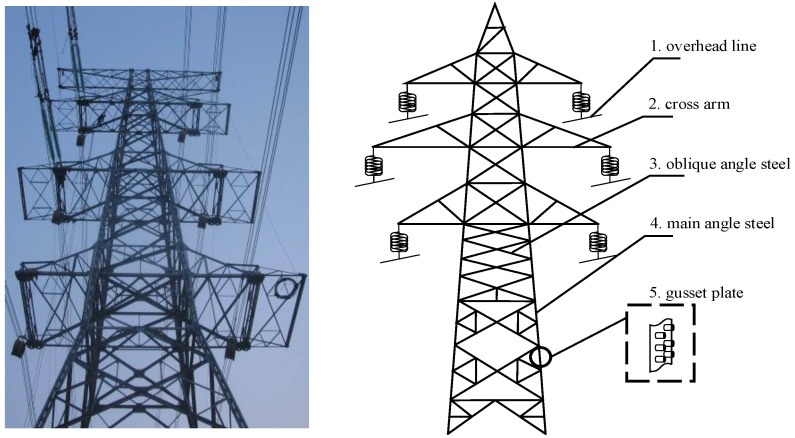
Transmission tower.

The role of transmission lines is to transmit and distribute electrical energy. As the support of the transmission line, the reliability of the transmission tower is an important factor to ensure the stability of the power system. As shown in Figure 1, the transmission tower is mainly composed of overhead line, cross arm, oblique angle steel, main angle steel, and gusset plate.

The structure of the transmission tower belongs to the space truss structure, which is composed of angle steel of various sizes, and the connection is fixed with bolts. The main angle steel of the transmission tower is mainly used as the support of the iron tower to improve the overall stability. The oblique angle steel adopts the form of staggered connection to support the main angle steel.

Through the analysis of the structure of the transmission tower, it can be seen that the complexity of the distribution of components makes it difficult for traditional positioning methods such as laser positioning and SLAM navigation to complete the precise positioning of the robot in such an environment. For the robots used for bolt tightening, the relative position information of these components in the working environment of the transmission tower remains basically invariant. The space size and feature points of the components are invariant in the Cartesian space coordinate system of the transmission tower [14]. Therefore, the position information of these feature points can be quantified as identifiable marker points, and the 3D space coordinates of these marker points can be read through the 3D information model of the transmission tower to assist in positioning.

There are two main methods for establishing a 3D information model of a transmission tower. The first method is to use lidar scanning technology to scan around the transmission tower to obtain the spatial 3D information of the transmission tower, and then use PointCloud, Cyclone and other software to perform 3D reconstruction. There is a large error between the model established in this way and the entity, which cannot meet the needs of precise positioning of the robot [15]. The second is to generate the geometric model through the complete 2D CAD drawings and lofting dimensions of the transmission tower provided by the design institute, adding the components to the tower model one by one, then adding the geometric dimension information of the components, and finally using the 3D model building software such as 3ds MAX to build up the components of the tower with 3D primitives, thereby establishing a complete 3D model of the transmission tower [16]. This modeling method realizes the connection between design and processing, and ensures the accuracy of 3D model information. This paper mainly establishes the 3D information model of the transmission tower by the second method to assist the robot to complete the positioning.

### 2.2. Deconstruction of the 3D Information Model of a Transmission Tower

**Figure 2:**
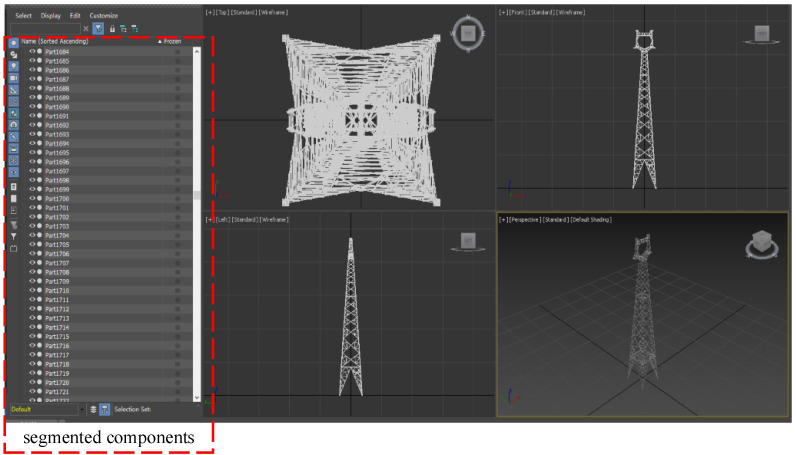
Analysis of the 3D information model of a transmission tower.

The positioning method proposed in this paper mainly uses the bolt vertexes in the transmission tower as the positioning beacons, and obtains the spatial position information of the beacons by deconstructing the 3D model file of the transmission tower through 3ds MAX software. Taking the 11-XJJ157-1D2SZ2-27-30.dae 3D model file provided by Electric Power Design Institute as an example, We first import the model into 3ds MAX software, and then set the coordinate system of the overall transmission tower model to coincide with the world coordinate system in 3ds MAX software. Finally, we use the “devide” command of 3ds MAX software to decompose the overall model of the transmission tower into components such as bolts and angle steel, keeping the relative position relationship unchanged. As shown on the left side of Figure 2, all components of the transmission tower model in 3ds MAX are split into individually selectable individuals.

**Figure 3:**
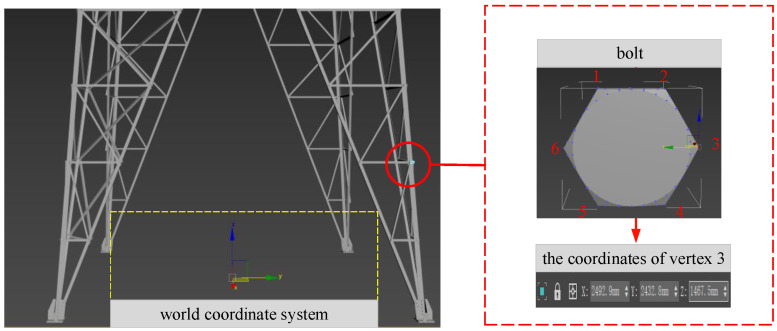
Coordinate information of the bolt vertices.

In the 3ds MAX software, the bolt assembly of the transmission tower can be selected and presented in the world coordinate system in an isolated manner, and each vertex of the bolt can be displayed through the function of cutting corners, so as to obtain its 3D space coordinates in the world coordinate system. In Figure 3, the vertices of the bolts hexagonal profile are marked, the upper left corner is marked as vertex 1, and the numbers of subsequent vertices increase clockwise from 2 to 6, so that the coordinates of the corresponding vertices of each bolt can be read like vertex 3 through the information model.

The climbing robot climbs along the legs of the transmission tower, so it is necessary to establish an information model database for the four legs of the transmission tower, respectively. First, we read the spatial coordinate information of the bolt vertexes from the 3D information model. Then, we assign a unique ID to each bolt, which starts from the bottom to the top along the ridge line of the angle steel. Finally, we store the 3D space coordinate information of the corresponding vertex of the bolt into the database.

## 3. Robot Pose Estimation

**Figure 4:**
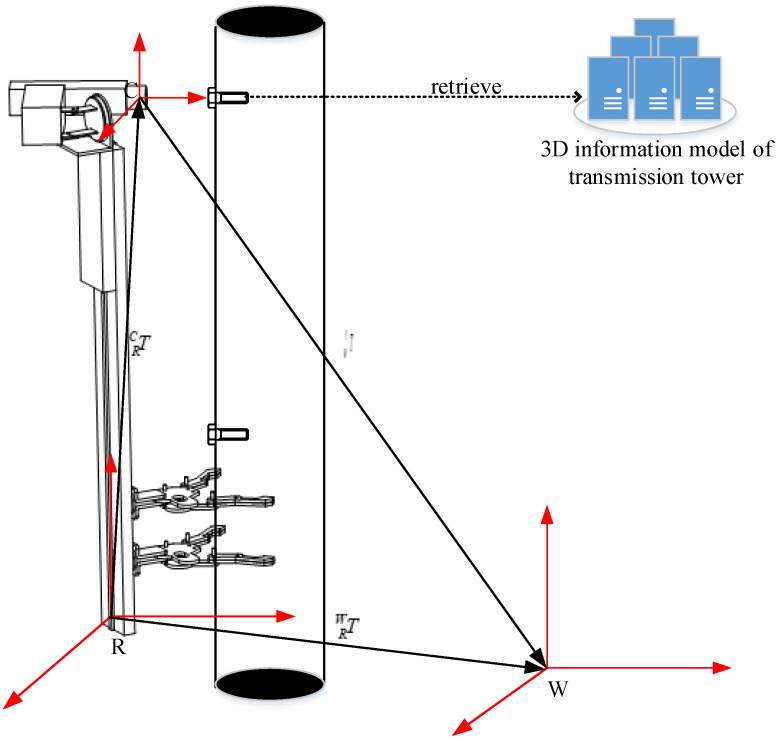
Schematic diagram of the robot pose solution.

The schematic diagram of the pose calculation process of the climbing robot is shown in Figure 4. The world coordinate system, the base coordinate system of the robot, and the camera coordinate system in the working environment of the transmission tower are represented by *W*, *R*, and *C*, respectively.

The pose estimation steps of the robot are as follows:

First, the robot performs real-time scanning and detection through the configured camera during the climbing process and calculates the ID information of the bolts in the current camera image sequentially.

In the second step, the pixel coordinate information of the bolt vertex in the image is recorded, and then the 3D space coordinate of the corresponding bolt vertex is recorded from the 3D information model of the transmission tower.

In the third step, the transformation matrix between the camera coordinate system and the working environment of the transmission tower coordinate system can be solved by using the method of point feature positioning, which is represented by TCW.

In the fourth step, the transformation matrix, which is represented by TRC, between the robot base coordinate system and the camera coordinate system, is solved by using the hand-eye calibration method [17].

In the last step, the above information is collected to perform the matrix operation, and the pose information of the robot in the coordinate system of the working environment of the transmission tower can be obtained. The pose information is represented by TRW:

(1) TRW=TCW×TRC

### 3.1. Extraction of the Bolt Edge Features

In the process of robot positioning, it is necessary to obtain the pose of the camera in the world coordinate system at first, and then calculate the current position information of the robot through the hand-eye calibration matrix of the robot and the camera.

Since the pose of the camera in the world coordinate system has been obtained above, it is only necessary to obtain the position information, that is, the pixel coordinates in the image. On account of the edge contour of the bolt being hexagonal, this feature can be used to extract the edge feature of the bolt in the image to obtain position information.

However, the image has factors such as inconspicuous edge information, noise, and low contrast, which makes it very difficult to directly extract the edge features of bolts without preprocessing. To accurately identify and record the pixel coordinates of the bolt vertices in the camera image, it is important to preprocess the image to optimize the information in the image.

**Figure 5:**
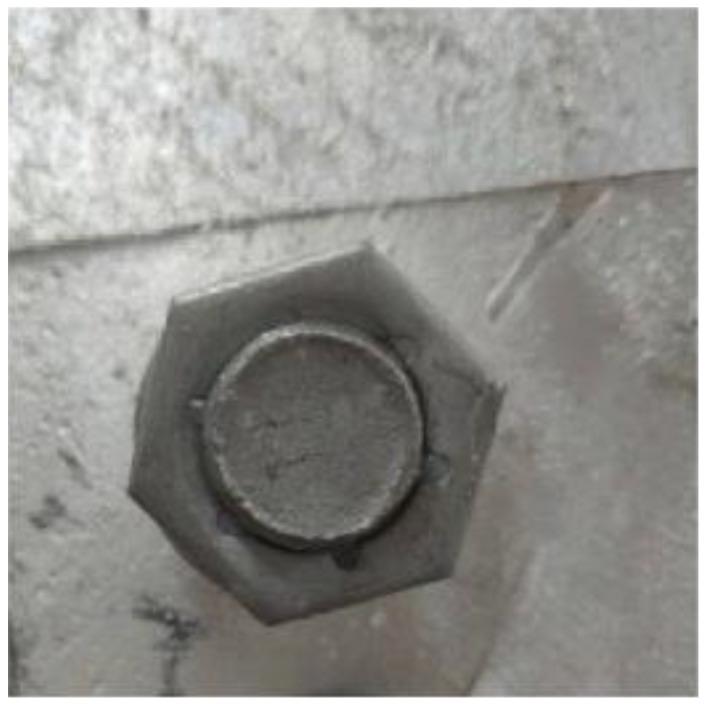
RGB image.

**Figure 6:**
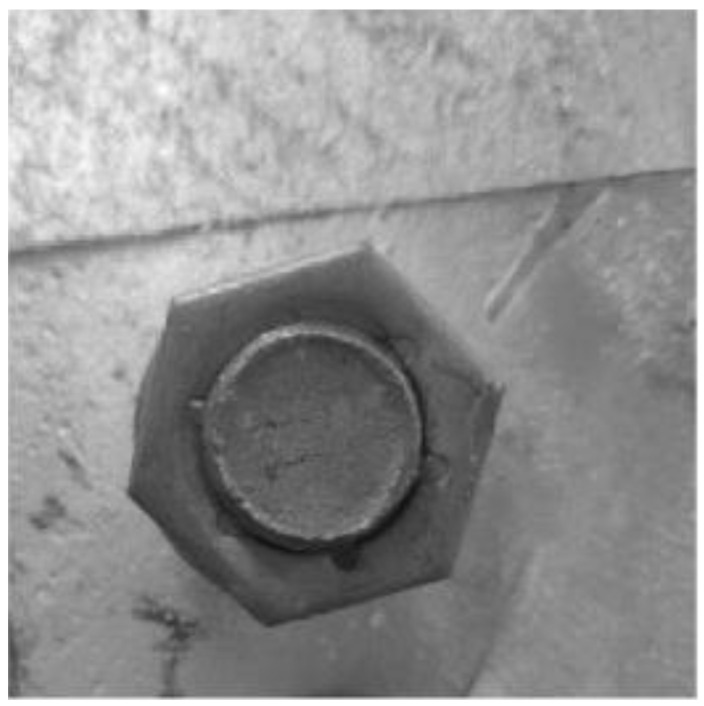
Grayscale image.

Therefore, the RGB image is converted into a grayscale image by the RGB2GRAY operator in OpenCV. Figure 5 shows the RGB image, and the Figure 6 shows the converted grayscale image.

**Figure 7:**
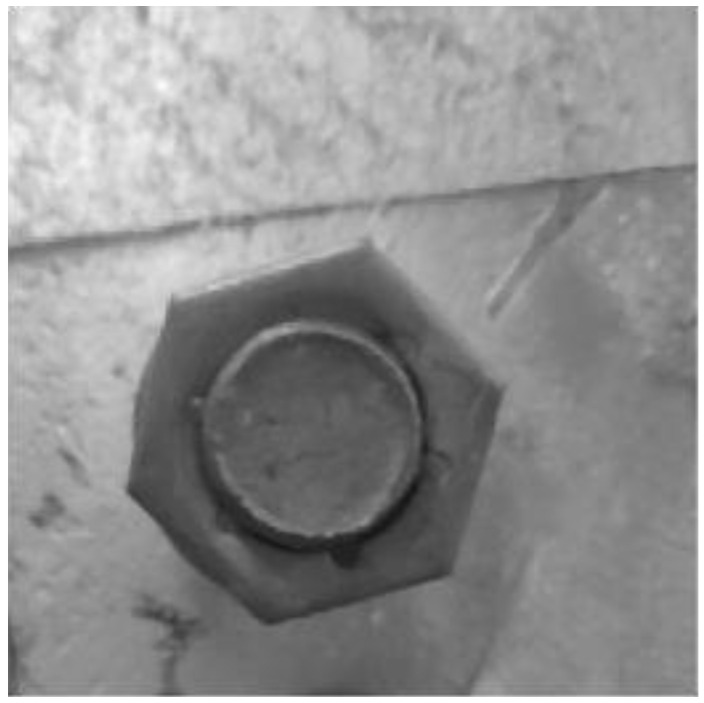
Denoising effect.

The main factor affecting image quality is noise, however, noise is mainly produced by two factors. The first factor is the interference of the bolt-fastening robot system, such as the noise caused by the rotation of the motor during climbing. The second factor is the interference of the camera’s own system, such as the thermal noise of the camera. It can be seen from the above that image denoising is very important in the image processing process, and a good denoising effect can improve the accuracy of bolt edge feature extraction. Therefore, this paper adopts the median filter method to solve the problem of image denoising, and the effect is shown in Figure 7.

Due to the special working environment of the transmission tower climbing robot, the background of collected image consists of main angle steel, oblique angle steel and so on, which makes the whole picture single in tone and low in contrast. This interference adds a certain degree of difficulty to the edge extraction of the hexagonal bolt.

**Figure 8:**
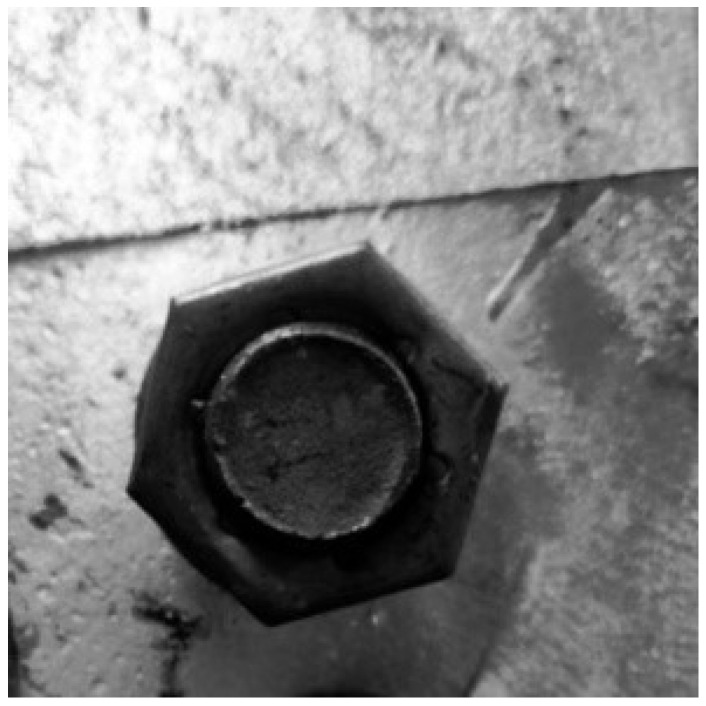
Image enhancement effect.

Therefore, it is necessary to perform enhancement processing on the collected image, so that the target object is more clearly presented in the image compared to the original image. Therefore, this paper uses the histogram equalization method to enhance the image, and the result is shown in Figure 8.

**Figure 9:**
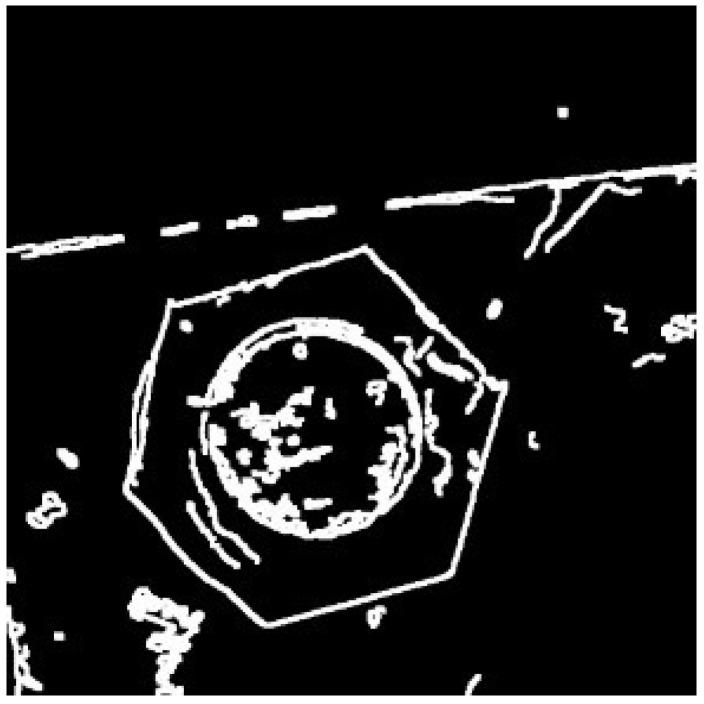
Edge detection result.

However, there is a high probability that the gray value of the pixel in the image will change abruptly at the edge of the image. It is well known that the Canny edge detector has an excellent edge detection effect. Therefore, in order to extract the edge features of the bolt hexagon contour better than the original image, this paper uses the Canny edge detector to effectively extract the edge of the denoised and enhanced image, and the processing result of the Canny edge detector is shown in Figure 9.

The basic steps are as follows:

1. Use the Gaussian filter to input the image, and take the convolution operation on the original image;
2. Use the finite difference of the first-order partial derivatives to calculate the gradient magnitude image and the angle image;
3. To exclude non-edge information, non-maximum suppression is performed on the gradient magnitude image;
4. Use dual threshold and connection analysis to detect the connection of edges. After many experiments and comparisons, the comprehensive level of the extraction effect is the best when the ratio of the high threshold and the low threshold is 3:1.

**Figure 10:**
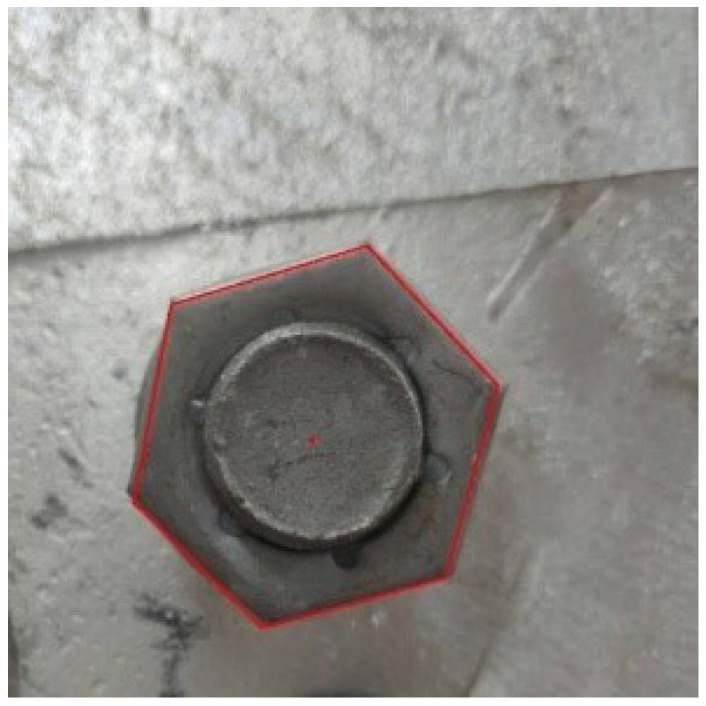
Edge feature extraction.

After the image is preprocessed, the Hough transform method is used to extract the hexagonal edge contour in the image. The hexagons that are closest to the bolt profile obtained experimentally are shown in Figure 10, and at the same time, the pixel coordinates of the hexagon vertices in the best image can also be recorded.

The pixel coordinates $\left\{u_{1},u_{2},\ldots ,u_{6}\right\}$ of the hexagonal contour vertices of the bolt in the camera image correspond to the 3D coordinates $\left\{c_{1},c_{2},\ldots ,c_{6}\right\}$ in the world coordinate system of the transmission tower working environment in turn to form a feature point pair, where $c_{i},u_{i}$ represents the *i*-th vertex of the hexagonal contour of the bolt.

### 3.2. Pose Estimation Based on Point Feature Localization

**Figure 11:**
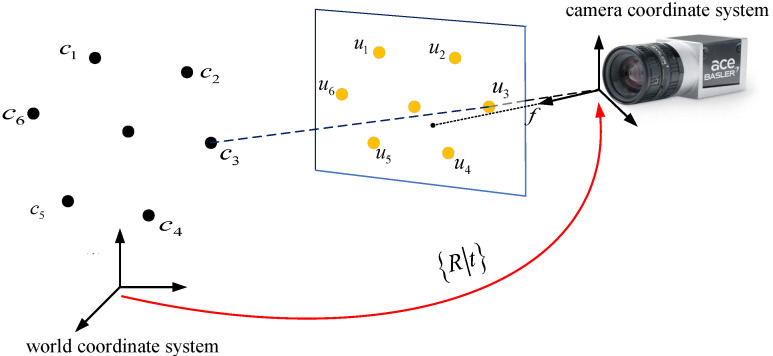
Schematic diagram of the PnP problem.

Knowing the spatial coordinates and image coordinates of n feature points, and how to calculate the pose of the camera in space is a classic PnP (Perspective-n-Point) problem. As shown in Figure 11, the goal of PnP is to get the relative pose between the object and the camera from a set of n pairs between 3D points and their corresponding 2D projections on the focal plane. It is a pose estimation proposed by Fishler in 1981, which is currently widely used in the fields of camera pose estimation [18], visual tracking [19] as well as recognition and grasping [20].

To sum up, the 3D coordinate $\left\{c_{1},c_{2},\ldots ,c_{6}\right\}$ of the bolt vertex in the world coordinate system of the transmission tower and the pixel coordinate $\left\{u_{1},u_{2},\ldots ,u_{6}\right\}$ in the camera image of the bolt can be formed into six sets of feature point pairs.

So it can be constructed as a least squares problem. By using the PnP algorithm to initially estimate the camera pose, and then using multiple sets of feature point pairs to continuously correct the error through an iterative method, an accurate camera pose can be obtained.

The six vertices *P* of the outline of the bolt in the 3D working space of the transmission tower correspond to the point *Q* on the camera imaging plane in turn. Let the coordinate of a certain space point be $P_{i}={\left[X_{i},Y_{i},Z_{i}\right]}^{T}$, and its pixel coordinate on the camera plane is $Q_{i}={\left[u_{i},v_{i}\right]}^{T}$. According to the principle of camera imaging, the relationship between the available pixel position and the 3D space point position is as follows:

(2) $$\begin{array}{c} z_{i}\left[\begin{array}{c} u_{i}\\ v_{i}\\ 1 \end{array}\right]=\left[\begin{array}{ccc} f_{x} & 0 & u_{0}\\ 0 & f_{y} & v_{0}\\ 0 & 0 & 1 \end{array}\right]\left[\begin{array}{cc} R_{3\times 3} & T_{3\times 1} \end{array}\right]\left[\begin{array}{c} X_{i}\\ Y_{i}\\ Z_{i}\\ 1 \end{array}\right]=K\left[\begin{array}{cc} R_{3\times 3} & T_{3\times 1} \end{array}\right]\left[\begin{array}{c} X_{i}\\ Y_{i}\\ Z_{i}\\ 1 \end{array}\right] \end{array}$$

where $\left[R_{3\times 3},T_{3\times 3}\right]$ represents the preliminary estimated camera pose solved by the PnP algorithm, which can be represented by the quaternion:

(3) $$\begin{array}{c} \left[\begin{array}{cc} R_{3\times 3} & T_{3\times 1} \end{array}\right]=\left[\begin{array}{cccc} \begin{array}{c} 1-2q_{2}^{2}-2q_{3}^{2}\\ 2q_{1}q_{2}-2q_{0}q_{3}\\ 2q_{1}q_{3}+2q_{0}q_{2} \end{array} & \begin{array}{c} 2q_{1}q_{2}+2q_{0}q_{3}\\ 1-2q_{1}^{2}-2q_{3}^{2}\\ 2q_{2}q_{3}-2q_{0}q_{1} \end{array} & \begin{array}{c} 2q_{1}q_{3}-2q_{0}q_{2}\\ 2q_{2}q_{3}+2q_{2}q_{1}\\ 1-2q_{1}^{2}-2q_{2}^{2} \end{array} & \begin{array}{c} t_{1}\\ t_{2}\\ t_{3} \end{array} \end{array}\right] \end{array}$$

where $q_{0}^{2}+q_{1}^{2}+q_{2}^{2}+q_{3}^{2}=1$, then formula (2) can be expressed as:

(4) $$\begin{array}{c} \left[\begin{array}{c} u_{i}\\ v_{i}\\ 1 \end{array}\right]=\frac{K}{z_{i}}\left[\begin{array}{cccc} \begin{array}{c} 1-2q_{2}^{2}-2q_{3}^{2}\\ 2q_{1}q_{2}-2q_{0}q_{3}\\ 2q_{1}q_{3}+2q_{0}q_{2} \end{array} & \begin{array}{c} 2q_{1}q_{2}+2q_{0}q_{3}\\ 1-2q_{1}^{2}-2q_{3}^{2}\\ 2q_{2}q_{3}-2q_{0}q_{1} \end{array} & \begin{array}{c} 2q_{1}q_{3}-2q_{0}q_{2}\\ 2q_{2}q_{3}+2q_{2}q_{1}\\ 1-2q_{1}^{2}-2q_{2}^{2} \end{array} & \begin{array}{c} t_{1}\\ t_{2}\\ t_{3} \end{array} \end{array}\right]\left[\begin{array}{c} X_{i}\\ Y_{i}\\ Z_{i}\\ 1 \end{array}\right]. \end{array}$$

We can simplify the formula by writing $Q_{i}={\left[\begin{array}{ccc} u_{i} & v_{i} & 1 \end{array}\right]}^{T}$, $T=\left[\begin{array}{cc} R_{3\times 3} & T_{3\times 1} \end{array}\right]$, $P_{i}={\left[\begin{array}{cccc} X_{i} & Y_{i} & Z_{i} & 1 \end{array}\right]}^{T}$, then, the equation above can be expressed as:

(5) $$\begin{array}{c} Q_{i}=\frac{1}{z_{i}}KTP_{i} \end{array}$$

However, in the actual calculation process, due to the influence of various factors, the equation is not completely established, and there will be an error between the real projection point of a 3D space point and the calculated pixel point obtained by the PnP algorithm, which is defined as the reprojection error.

(6) $$\begin{array}{c} E\left(q_{0},q_{1},q_{2},q_{3},t_{1},t_{2},t_{3}\right)=\sum_{i=1}^{6}e_{i}\left(q_{0},q_{1},q_{2},q_{3},t_{1},t_{2},t_{3}\right) \end{array}$$

(7) $$\begin{array}{c} e_{i}\left(q_{0},q_{1},q_{2},q_{3},t_{1},t_{2},t_{3}\right)=∥Q_{i}-\frac{1}{z_{i}}KTP_{i}∥ \end{array}$$

Formula (6) is constructed as the objective function of the nonlinear least squares optimization problem under constraints:

(8) $$\begin{array}{c} \xi^{*}=\arg\min_{\xi }\frac{1}{2}\sum_{i=1}^{6}{∥Q_{i}-\frac{1}{z_{i}}KTP_{i}∥}_{2}^{2} \end{array}$$

The camera pose preliminarily estimated by the PnP algorithm is used as the initial value, and then the Levenberg–Marquardt iterative algorithm is used to solve the parameter value corresponding to the minimum overall reprojection error. Next, we substitute the parameter value into Formula (3) to obtain the final pose transformation matrix $\left\{R|t\right\}$ between the camera coordinate system and the world coordinate system. Finally, the hand-eye calibration matrix of the integrated camera coordinate system and the robot base coordinate system can solve the position information of the climbing robot in the working environment of the transmission tower.

Furthermore, the bolt positioning process of the climbing robot is as follows:

In the first step, before the climbing robot starts to climb the tower, we set the initial pose of the robot to ensure that the camera can face the side of the angle steel of the transmission tower in a positive direction so that the camera can scan the bolts close to the edge of the angle steel. At the same time, the pose of the camera relative to the robot remains unchanged during the climbing process.

In the second step, the robot starts to climb along one leg of the transmission tower. During the process of climbing the tower, the camera performs real-time scanning and detection, collects image information, and transmits the information to the terminal system.

In the third step, we read the ID information of the bolt marking point closest to the initial climbing position of the robot, and record it as (ID)init. Then, every time the camera detects the bolt marking point during the tower climbing process, we add one to the previous bolt mark point ID and record it as the current bolt mark point ID. Next, we retrieve the 3D space coordinates of the current bolt vertex through the ID information in the transmission tower information model database. Finally, the pixel coordinates of the vertices in the visual information are fused to calculate the pose transformation matrix of the camera coordinate system and the world coordinate system using the method of point feature positioning.

In the last step, the transformation matrix of the camera coordinate system and the robot base coordinate system is calculated using the method of hand-eye calibration. Then, the transformation matrix is used to calculate the position of the robot in the working environment of the transmission tower.

**Figure 12:**
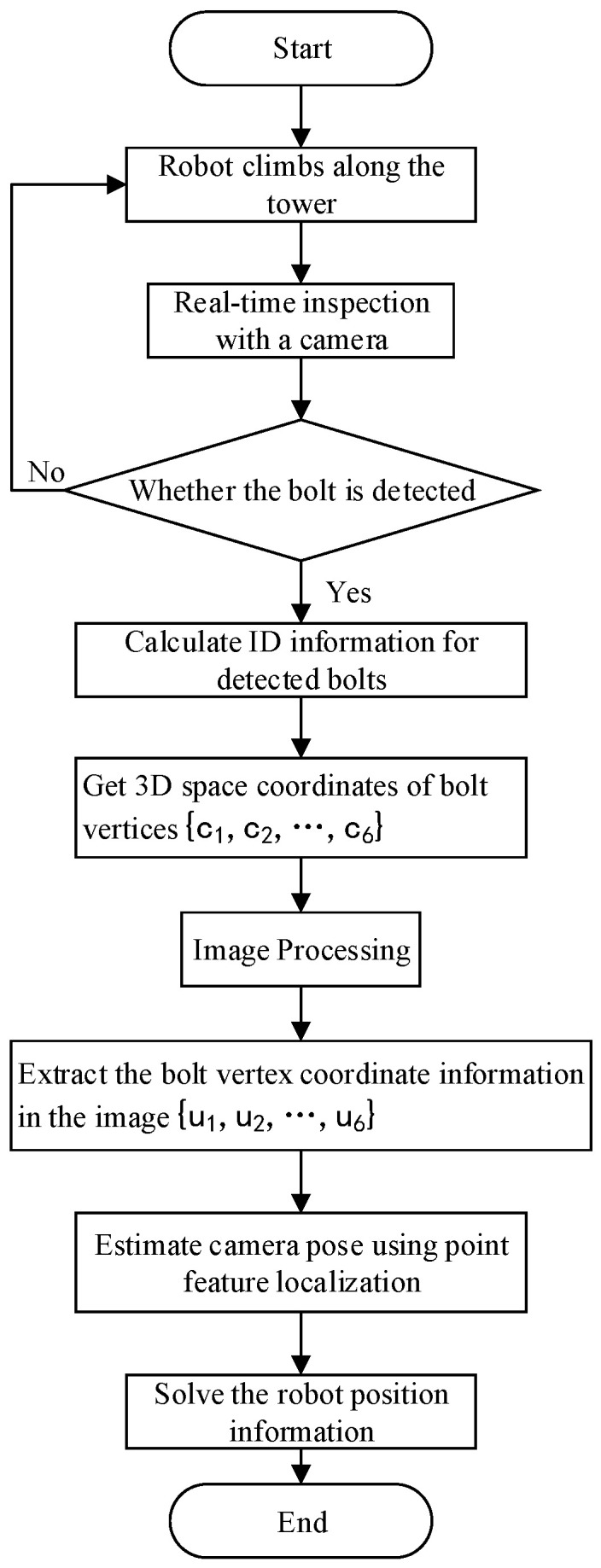
Positioning flow chart.

The overall flow of the bolt positioning method is shown in Figure 12.

## 4. Experiments and Analysis

The camera used in the positioning experiment is a Basler industrial camera, model acA2500-14gc (CS-Mount). In this experiment, the calibration tool based on the Zhang Zhengyou calibration method [21] integrated in the MATLAB toolbox is used to complete the camera calibration experiment, and the calculated internal parameter matrix *K* of the camera is:

(9) $$\begin{array}{c} K=\left[\begin{array}{ccc} f_{x} & 0 & c_{x}\\ 0 & f_{y} & c_{y}\\ 0 & 0 & 1 \end{array}\right]=\left[\begin{array}{ccc} 955.1682 & 0 & 636.2796\\ 0 & 955.1012 & 439.1885\\ 0 & 0 & 1 \end{array}\right] \end{array}$$

The distortion parameters are:

(10) $$\begin{array}{c} D=\left\{k_{1},k_{2},p_{1},p_{2}\right\}=\left\{-0.0676,0.0908,1.8273\times 10^{-4},-1.2887\times 10^{-4}\right\} \end{array}$$

**Figure 13:**
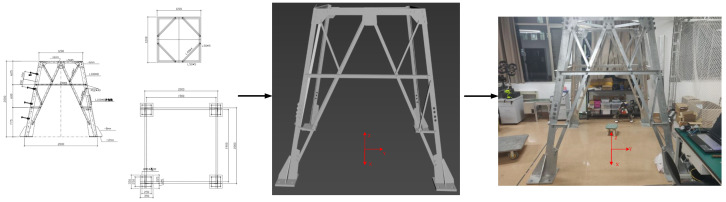
Transmission tower experimental environment.

In order to verify the feasibility of the positioning method proposed in this paper, a 2-meter-high transmission tower working environment is built in the laboratory. Due to the limited space in the laboratory, only the part below the cross arm of the transmission tower was built. For the sake of the accuracy of the information model of each component, the 3D information model of the transmission tower and the tower entity are built synchronously based on the method of the 2D CAD drawing of the transmission tower, as shown in Figure 13.

**Figure 14:**
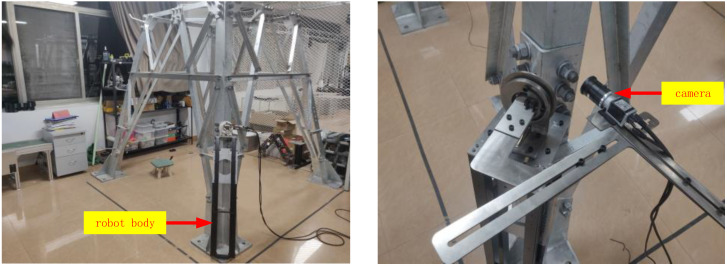
Positioning experiment platform.

In the experiment, the fixing method of the camera is shown in Figure 14. The three-degree-of-freedom camera bracket is installed above the robot body, whose physical size is known through the design parameters, and the camera is fixed at the end of the camera bracket to form a positioning experiment platform. The transformation matrix TCR, the camera coordinate system, and the base coordinate system of the experiment platform can be obtained by calibration:

(11) TCR=0.05820.70730.7074263.220.0051−0.70710.7072181.340.99830.0001−0.01401081.980001

**Figure 15:**
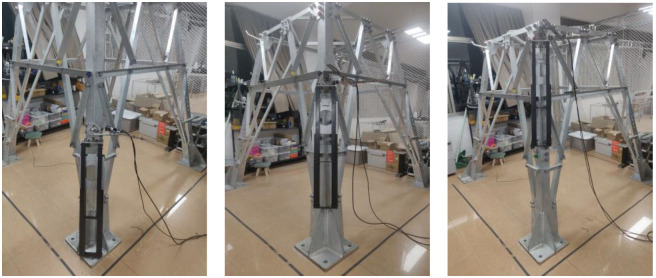
Acquisition of image information.

The experiment is divided into three groups. The bolts with heights of 100 cm, 130 cm and 180 cm on a single leg of the tower are selected as auxiliary positioning points, and the 3D space coordinates of the corresponding bolt vertices can be read from the tower information model. During the experiment, the experimental platform is fixed to the transmission tower at different heights, as shown in Figure 15, so that the camera can shoot the bolt auxiliary positioning points at different heights, and the distance between the camera and the bolt marking surface is kept at 3 cm.

**Figure 16:**
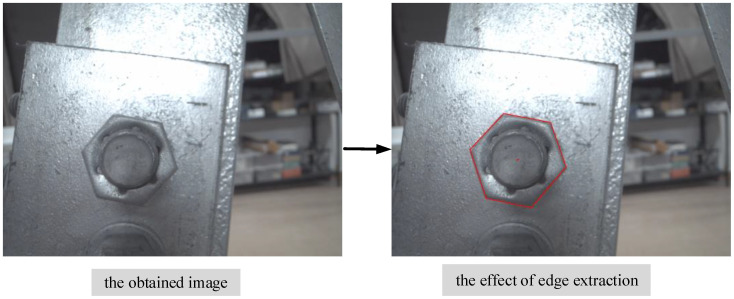
Bolt edge feature extraction.

In the positioning experiment, an industrial camera is used to capture the bolt image and preprocess the image through the above image processing method.The pixel coordinates of the vertices of the bolt hexagon contour can be read by performing edge feature extraction on the bolt contour in the image. Taking the bolt auxiliary positioning point with the height of 100 cm as an example, Figure 16 shows the image captured by the camera and the extraction results of the bolt contour edge feature.

By obtaining the pixel coordinates of the bolt vertices and synthesizing the 3D space coordinates of the bolt vertices read from the 3D information model of the transmission tower, several 2D–3D feature point pairs can be formed. Based on the camera pose estimation results of the aforementioned feature point positioning method, the position information of the experimental platform can be calculated, synthesizing the transformation matrix of the camera coordinate system and the experimental platform base coordinate system. The positioning experiments are carried out at three bolt-assisted positioning points with different heights, and the measurements are repeated 10 times at the 3 positions. Using the positioning method proposed in this paper, the deviations between the position information of the experimental platform and the actual physical measurement position information are calculated respectively. We take the maximum values and average values of the position deviations in the 10 test results, and the results are shown in Table 1.

According to the results of the positioning experiments, it can be seen that the accuracy of the positioning method is within 1 cm. It is worth noting that the positioning accuracy of different heights is basically the same, and the accumulated error will not be caused by the continuous increase of the climbing height.

According to the position deviation of the experiment data and the height of the transmission tower, the relative deviation in this experiment is listed, and the three other methods introduced in the previous article are compared. The results are shown in Table 2.

It can be seen from Table 2 that the positioning method based on the 3D model of the transmission tower and visual sensor has higher accuracy. The experiment proves the rationality and superiority of the positioning method of the transmission tower.

## 5. Summary

Aiming at the positioning problem of the climbing robot in the working environment of the transmission tower, this paper proposes an intelligent positioning method that integrates the 3D information model of the tower and the visual information. Firstly, the 3D information model of the transmission tower and the spatial coordinate information of its internal bolt feature vertices are analyzed; then the pixel coordinate information of the bolt mark vertices in the camera image is obtained through image processing; finally, the feature point positioning method is used for the pose estimation of the camera and the pose calculation of the climbing robot, synthesizing the 3D space coordinates and pixel coordinates of the bolt mark vertices.

The results of the positioning experiment show that the positioning method can assist the climbing robot to complete the autonomous positioning in the working environment of the transmission tower, and the positioning accuracy is within 1 cm.

## Figures and Tables

**Table 1:** Results of positioning experiments.

Position (Height)	Value	X Deviation (cm)	Y Deviation (cm)	Z Deviation (cm)
100 cm	Maximum value	0.716	0.734	0.661
	Average value	0.636	0.625	0.581
130 cm	Maximum value	0.695	0.656	0.624
	Average value	0.642	0.584	0.587
180 cm	Maximum value	0.732	0.691	0.683
	Average value	0.675	0.637	0.615

**Table 2:** Relative deviation comparison results.

	SGI	VRL	CVIS	Ours
Maximum relative deviation	2.8%	1.5%	6.0%	0.61%
Mean relative deviation	1.4%	0.63%	3.6%	0.54%

## Data Availability

Not applicable.
